# Association between aggregate index of systemic inflammation and in-hospital new-onset AF in myocardial infarction with nonobstructive coronary arteries: A retrospective cohort study

**DOI:** 10.1097/MD.0000000000049760

**Published:** 2026-07-17

**Authors:** Qian Shen, Yifei Tao, Jiayu Yin, Zhixian Chen, Yu Qian, Kaili Yang

**Affiliations:** a Department of Cardiology, The Affiliated Changshu Hospital of Nantong University, Changshu, Jiangsu, China; b Department of Cardiology, The Second Affiliated Hospital of Soochow University, Suzhou, Jiangsu, China.

**Keywords:** aggregate index of systemic inflammation, coronary microvascular dysfunction, MINOCA, new-onset atrial fibrillation, risk stratification

## Abstract

In recent years, with a growing understanding of coronary microvascular dysfunction, myocardial infarction with nonobstructive coronary arteries (MINOCA) has been proposed as a distinct type of myocardial infarction. The management of atrial fibrillation (AF) coexisting with myocardial infarction remains a major challenge in clinical practice. This study aims to explore the association between the inflammatory marker aggregate index of systemic inflammation (AISI) and new-onset AF (NOAF) in patients with MINOCA. In this single-center, retrospective study, we consecutively enrolled patients with MINOCA from January 2019 to June 2025. AISI was calculated as (Neutrophil count × Platelet count × Monocyte count)/Lymphocyte count from procedural complete blood count. NOAF was defined as new-onset AF after admission in patients with no previous history of AF. Multivariable logistic regression was employed to screen for factors associated with NOAF. Restricted cubic spline was used to characterize the dose-response relationships between AISI and NOAF. Receiver operating characteristic curves were constructed to evaluate the discriminative performance of AISI. Among 409 patients with MINOCA, 38 (9.3%) developed NOAF. In multivariable analysis, AISI (odds ratio 2.335, 95% confidence interval [CI] 1.532–3.560, *P* < .001) and C-reactive protein (odds ratio 1.009, 95% CI 1.002–1.017, *P* = .015) remained independently associated with NOAF, which suggests that AISI provides additional information independent of the traditional inflammatory marker C-reactive protein in relation to NOAF. Restricted cubic spline analysis suggested an initial nonlinear dose-response relationship between AISI and NOAF in the unadjusted model; however, this association was no longer statistically significant after adjustment for relevant clinical covariates. In receiver operating characteristic analysis, AISI yielded an area under the curve of 0.712 with an optimal cutoff of 750 (sensitivity 0.737, specificity 0.650, 95% CI 0.617–0.808, *P* < .001). Higher AISI is independently associated with in-hospital NOAF in patients with MINOCA, although its discriminative performance is moderate, suggesting that AISI may serve as an adjunctive rather than a standalone risk marker.

## 1. Introduction

With growing recognition of coronary microvascular dysfunction (CMD), myocardial infarction with nonobstructive coronary arteries (MINOCA) has attracted increasing attention as a distinct subtype of myocardial infarction.^[1]^ Clinically, MINOCA is characterized by the absence of significant stenosis in the major epicardial coronary arteries on angiography, and microvascular dysfunction is one of its key pathophysiological mechanisms. Notably, the prognosis of MINOCA is comparable to that of obstructive myocardial infarction.^[2,3]^ New-onset atrial fibrillation (AF) (NOAF), a common complication of acute myocardial infarction (MI) (AMI), has been shown to be closely associated with major adverse cardiovascular events.^[4,5]^ The challenge, however, is that there remains no satisfactory strategy that is both effective and safe for managing AF in the setting of myocardial infarction.^[6]^ Therefore, identifying these high-risk patients and optimizing risk stratification is of substantial clinical value.

In the acute phase of MI, systemic inflammatory activation, enhanced platelet reactivity, and microvascular perfusion abnormalities are common.^[7]^ Inflammatory mediators can alter the expression of atrial ion channels and activate atrial fibroblasts, leading to electrical and structural remodeling that provides the pathological substrate for the development of AF.^[8,9]^ The aggregate index of systemic inflammation (AISI), a composite inflammatory score integrating neutrophil, lymphocyte, monocyte, and platelet counts, theoretically captures a broader spectrum of inflammation-thrombosis biology than single markers or ratios.^[10–12]^ In a retrospective cohort of 1044 AMI patients, higher AISI levels were independently associated with increased risks of major adverse cardiovascular events, suggesting AISI as a useful early prognostic marker for adverse outcomes in AMI.^[13]^ In a case-control study of 91 paroxysmal AF (PAF) and 97 non-PAF participants, the level of AISI was positively correlated with the incidence of PAF.^[14]^ In MINOCA, mechanisms such as CMD, plaque disruption, vasospasm, and thrombosis are all influenced by inflammatory and immune pathways.^[15]^ Hence, AISI could provide a more comprehensive representation of the systemic inflammatory burden involved in MINOCA pathophysiology. Previous studies have demonstrated that some systemic inflammation indices are associated with the development of AF, including post–myocardial infarction AF.^[16,17]^ However, the relationship between AISI and NOAF in patients with MINOCA remains unclear.

This study aims to determine the incidence of NOAF in patients with MINOCA and to evaluate the association between AISI levels and the occurrence of NOAF during hospitalization. We hypothesized that higher admission AISI is independently associated with in-hospital NOAF among MINOCA patients.

## 2. Methods

### 2.1. Study population

**Figure 1.**
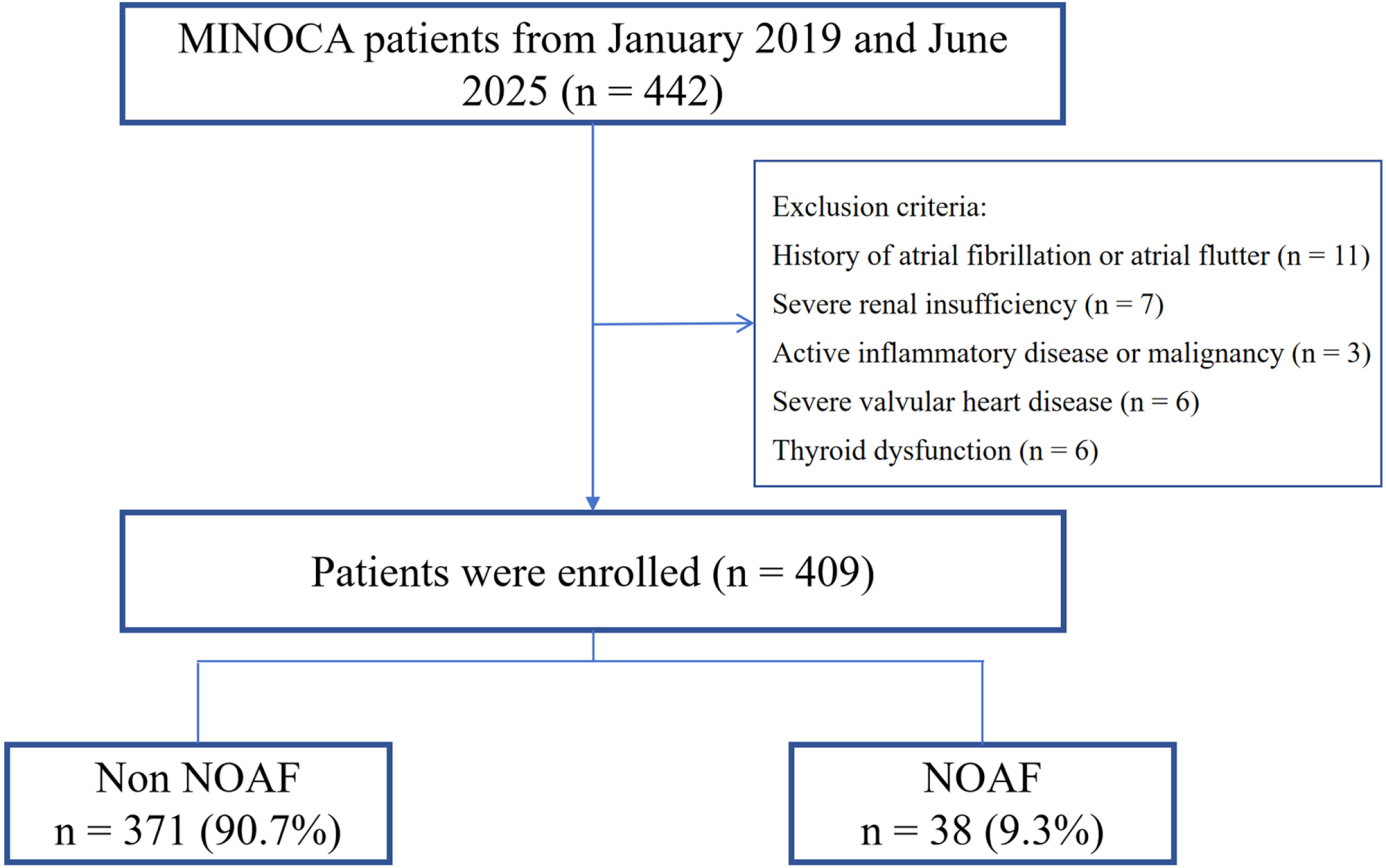
The study flowchart. MINOCA = myocardial infarction with nonobstructive coronary arteries, n = number of participants, NOAF = new-onset atrial fibrillation.

This single-center, retrospective study consecutively enrolled patients hospitalized at the Second Affiliated Hospital of Soochow University between January 2019 and June 2025 who were ultimately diagnosed with MINOCA. The study protocol was approved by the Ethics Committee of the Second Affiliated Hospital of Soochow University and complied with the Declaration of Helsinki (NO. JD-HG-2025-105). Given the retrospective analysis of existing medical records and the absence of additional interventions, the requirement for written informed consent was waived. Inclusion criteria were: age ≥ 18 years; presentation consistent with the diagnostic framework for AMI^[18]^; coronary angiography demonstrating no ≥ 50% stenosis in any major epicardial coronary segment; a diagnosis of MINOCA per the European Society of Cardiology working definition after exclusion of overt nonischemic causes of myocardial injury^[19]^; A minimum of 72 hours of continuous electrocardiogram (ECG) monitoring (telemetry) and at least 2 12-lead ECGs; Availability of complete clinical data. Exclusion criteria were: history of AF or atrial flutter (based on medical history, ECGs, and medical records); severe renal insufficiency (estimated glomerular filtration rate [eGFR] < 30 mL·min^−1^·1.73 m^−2^); active inflammatory disease or malignancy; severe valvular heart disease; thyroid dysfunction. Finally, a total of 409 patients were included in the final analysis (Fig. 1).

### 2.2. Data collection

Baseline characteristics and risk factors were extracted from the electronic medical record system, including age, sex, smoking status, hypertension, diabetes, and ST-segment elevation on presentation. Complete blood count obtained prior to coronary angiography was recorded. AISI was calculated as follows:


$$AISI=\frac{Neutrophil\;\;\;count\;\;\;\times \;\;\;Platelet\;\;\;count\;\;\;\times \;\;\;Monocyte\;\;\;count}{Lymphocyte\;\;\;count}$$

Additional in-hospital laboratory data collected within the same timeframe included fasting blood glucose (FBG), glycated hemoglobin (hemoglobin A1c), total cholesterol, triglycerides, eGFR, and markers of inflammation and cardiac injury or stress: peak C-reactive protein (CRP), peak troponin T, and peak N-terminal pro-B-type natriuretic peptide. To address right-skewness and heteroscedasticity and better satisfy regression assumptions, natural logarithmic transformations were applied to AISI, troponin T, and N-terminal pro-B-type natriuretic peptide when these variables were modeled as continuous predictors. Left ventricular ejection fraction was obtained from contemporaneous echocardiography reports. AF was identified on electrocardiography by the absence of distinct P waves and the presence of irregular fibrillatory activity with varying morphology and amplitude, together with a fully irregular ventricular response. The diagnosis required documentation on a 12-lead ECG or a single-lead recording lasting at least 30 seconds.^[20]^ NOAF was defined as new-onset AF after admission in patients with no previous history of AF.

### 2.3. Statistical analysis

All statistical analyses were performed using SPSS version 27.0 (IBM Corp.) and R version 4.3 (R Foundation for Statistical Computing). Normality of continuous variables was assessed with the Shapiro–Wilk test. Normally distributed variables are presented as mean ± standard deviation and compared using the *t*-test; non-normally distributed variables are presented as median (interquartile range) and compared using the Mann–Whitney *U* test. Categorical variables are presented as counts (percentages) and compared using the *χ*^2^ test or Fisher exact test, as appropriate. All variables were initially entered into univariable logistic regression to screen for factors potentially associated with NOAF. To mitigate collinearity and redundancy, variables with (*P* < .05) in univariable analysis (excluding the individual leukocyte and platelet components [neutrophil, platelet, monocyte, and lymphocyte counts]) and traditional risk factors were subsequently included in a multivariable logistic regression model using a stepwise selection procedure. Multicollinearity among all candidate variables entered into the multivariable logistic regression model was assessed using the variance inflation factor (VIF). A VIF value > 5 was considered indicative of potentially relevant multicollinearity. No candidate variable had a VIF > 5 in the candidate model. Restricted cubic spline (RCS) (4 knots: 5th, 35th, 65th, 95th) functions were used to characterize the dose-response relationships between AISI and the risk of NOAF. Receiver operating characteristic (ROC) curves were constructed to evaluate the discriminative performance of AISI for NOAF. All tests were 2-sided, and statistical significance was set at (*P* < .05).

## 3. Results

### 3.1. Patient characteristics

Among 409 MINOCA patients, 38 (9.3%) had concomitant NOAF. Patients who developed NOAF had significantly higher AISI [1148.29 (718.75–1987.73) vs 550.63 (285.19–961.96), *P* < .001], white blood cell (WBC), neutrophil count, and CRP, along with a lower lymphocyte count, compared with those without NOAF. Additionally, eGFR was lower and FBG was higher in the NOAF group (all *P* < .05) (Table 1).

**Table 1:** Baseline characteristics of study cohort.

Variables	NOAF (n = 38)	Non-NOAF (n = 371)	*P* value
Female, n (%)	15 (39.5)	171 (46.1)	.496
Age, yrs	69.55 ± 10.97	65.34 ± 13.78	.068
BMI, kg/m^2^	25.32 ± 3.85	24.55 ± 3.42	.187
Heart rate, bpm	80.58 ± 13.05	78.98 ± 14.22	.507
SBP, mm Hg	128.89 ± 20.97	127.30 ± 21.19	.658
DBP, mm Hg	82.11 ± 14.94	78.47 ± 14.10	.158
Smoking, n (%)	11 (28.9)	151 (40.7)	.169
Diabetes mellitus, n (%)	10 (26.3)	69 (18.6)	.251
Hypertension, n (%)	21 (55.3)	191 (51.5)	.734
ST elevation, n (%)	9 (23.7)	101 (27.2)	.639
AISI	1148.29 (718.75, 1987.73)	550.63 (285.19, 961.96)	< .001
WBC, ×10^9^/L	12.15 (9.30, 13.93)	9.70 (8.00, 12.10)	< .001
Neutrophil count, ×10^9^/L	10.41 (7.78, 12.27)	7.35 (5.54, 10.13)	< .001
Lymphocyte count, ×10^9^/L	1.10 (0.90, 1.60)	1.40 (1.00, 2.00)	.005
Monocyte count, ×10^9^/L	0.57 (0.38, 0.93)	0.51 (0.37, 0.70)	.139
Platelet count, ×10^9^/L	215.00 (185.25, 246.75)	211.00 (179.00, 252.00)	.826
CRP, mg/L	9.35 (3.95, 36.38)	2.40 (0.60, 9.10)	< .001
eGFR, mL/min/1.73 m^2^	97.46 ± 20.06	104.94 ± 20.01	.029
FBG, mmol/L	8.25 ± 3.89	6.85 ± 2.80	.005
TC, mmol/L	4.05 (3.51, 4.62)	4.29 (3.67, 4.99)	.203
TG, mmol/L	1.29 (0.80, 1.70)	1.23 (0.90, 1.71)	.861
HDL cholesterol, mmol/L	0.95 (0.87, 1.14)	0.97 (0.84, 1.16)	.975
LDL cholesterol, mmol/L	2.50 (2.02, 2.87)	2.65 (2.06, 3.20)	.184
HbA1c, %	6.63 ± 1.56	6.23 ± 1.42	.095
Troponin T, pg/mL	251.50 (95.55, 619.38)	235.60 (71.25, 681.50)	.651
NT-proBNP, pg/mL	485.59 (197.25, 1422.00)	393.40 (120.10, 1192.00)	.186
Na^+^, mmol/L	139.32 ± 5.80	139.86 ± 3.31	.377
K^+^, mmol/L	4.06 ± 0.51	3.96 ± 0.47	.228
Ca^+^, mmol/L	2.25 ± 0.16	2.22 ± 0.13	.224
LVEF, %	51.26 ± 6.14	50.95 ± 7.12	.791
Left atrial diameter, mm	40.74 ± 7.92	39.22 ± 6.35	.172
Length of stay, d	5.55 ± 1.67	6.11 ± 2.20	.132
Statins, n (%)	35 (92.1)	326 (87.9)	.600
ACEI/ARB, n (%)	24 (63.2)	184 (49.6)	.127
CCB, n (%)	15 (39.5)	118 (31.8)	.365
Beta-blockers, n (%)	26 (68.4)	224 (60.4)	.385
Aspirin, n (%)	34 (89.5)	307 (82.7)	.365
Clopidogrel/Ticagrelor, n (%)	33 (86.8)	304 (81.9)	.654

ACEI = angiotensin-converting enzyme inhibitor, AISI = aggregate index of systemic inflammation, ARB = angiotensin receptor blocker, BMI = body mass index, CCB = calcium-channel blocker, CRP = C-reactive protein, DBP = diastolic blood pressure, eGFR = estimated glomerular filtration rate, FBG = fasting blood glucose, HbA1c = hemoglobin A1c, HDL = high-density lipoprotein, LDL = low-density lipoprotein; LVEF = left ventricular ejection fraction, n = number of participants, NOAF = new-onset atrial fibrillation, NT-proBNP = N-terminal pro–B-type natriuretic peptide, SBP = systolic blood pressure, TC = total cholesterol, TG = triglycerides, WBC = white blood cell.

### 3.2. Logistic regression analysis of NOAF

**Figure 2.**
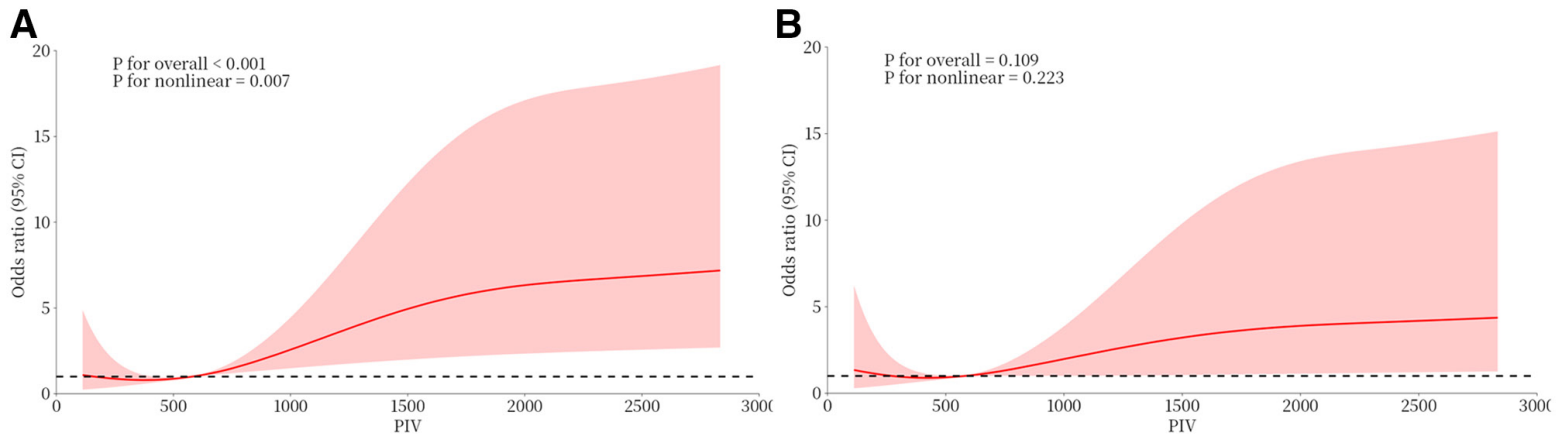
Dose-response relationship between inflammatory biomarkers and NOAF. (A) An unadjusted dose-response relationship between AISI and NOAF; (B) an adjusted dose-response relationship between AISI and NOAF. AISI = aggregate index of systemic inflammation, CI = confidence interval, NOAF = new-onset atrial fibrillation, OR = odds ratio.

Among baseline variables in univariable logistic regression (Table 2), a higher AISI was associated with NOAF (odds ratio [OR] 2.492, 95% confidence interval [CI] 1.647–3.769, *P* < .001), as were WBC (OR 1.198, 95% CI 1.091–1.316, *P* < .001), neutrophil count (OR 1.083, 95% CI 1.005–1.168, *P* = .036), CRP (OR 1.012, 95% CI 1.005–1.019, *P* = .001), a lower lymphocyte count (OR 0.592, 95% CI 0.363–0.964, *P* = .035), a lower eGFR (OR 0.984, 95% CI 0.970–0.999, *P* = .031), and higher FBG (OR 1.131, 95% CI 1.033–1.238, *P* = .008). The variables with *P* < .05) in univariable analysis and traditional risk factors were subsequently included in a multivariable logistic regression model using a stepwise selection procedure. The results showed that both AISI (OR 2.335, 95% CI 1.532–3.560, *P* < .001) and CRP (OR 1.009, 95% CI 1.002–1.017, *P* = .015) remained significantly associated with NOAF (Table 3). RCS analysis indicated a nonlinear dose-response relationship between AISI and NOAF (*P* for overall < .001, *P* for nonlinear = .007), while no statistically significant differences were detected after adjustment for relevant covariates (*P* for overall = .109, *P* for nonlinear = .223) (Fig. 2).

**Table 2:** Univariate regression analysis.

Variables	OR	95% CI	*P* value
Female, n (%)	0.763	0.386–1.508	.436
Age, yrs	1.025	0.998–1.052	.070
BMI, kg/m^2^	1.065	0.970–1.169	.187
Heart rate, bpm	1.008	0.985–1.032	.506
SBP, mm Hg	1.004	0.988–1.019	.658
DBP, mm Hg	1.018	0.995–1.042	.133
Smoking, n (%)	0.594	0.286–1.233	.162
Diabetes mellitus, n (%)	1.563	0.725–3.369	.254
Hypertension, n (%)	1.164	0.595–2.277	.657
ST elevation, n (%)	0.830	0.380–1.813	.640
AISI	2.492	1.647–3.769	< .001
WBC, × 10^9^/L	1.198	1.091–1.316	< .001
Neutrophil count, ×10^9^/L	1.083	1.005–1.168	.036
Lymphocyte count, ×10^9^/L	0.592	0.363–0.964	.035
Monocyte count, ×10^9^/L	2.005	0.963–4.173	.063
Platelet count, ×10^9^/L	1.001	0.995–1.007	.760
CRP, mg/L	1.012	1.005–1.019	.001
eGFR, mL/min/1.73 m^2^	0.984	0.970–0.999	.031
FBG, mmol/L	1.131	1.033–1.238	.008
TC, mmol/L	0.823	0.580–1.169	.277
TG, mmol/L	0.929	0.637–1.354	.701
HDL cholesterol, mmol/L	1.043	0.267–4.071	.952
LDL cholesterol, mmol/L	0.752	0.501–1.128	.169
HbA1c, %	1.181	0.970–1.437	.099
Troponin T, pg/mL	1.081	0.846–1.381	.534
NT-proBNP, pg/mL	1.169	0.969–1.409	.102
Na^+^, mmol/L	0.960	0.876–1.051	.375
K^+^, mmol/L	1.460	0.787–2.710	.230
Ca^+^, mmol/L	5.173	0.367–72.884	.223
LVEF, %	1.007	0.959–1.056	.791
Left atrial diameter, mm	1.033	0.986–1.083	.173
Length of stay, d	0.864	0.717–1.041	.123
Statins, n (%)	1.610	0.476–5.453	.444
ACEI/ARB, n (%)	1.742	0.874–3.473	.115
CCB, n (%)	1.398	0.704–2.777	.338
Beta-blockers, n (%)	1.422	0.696–2.906	.335
Aspirin, n (%)	1.772	0.608–5.168	.295
Clopidogrel/Ticagrelor, n (%)	1.455	0.548–3.864	.452

ACEI = angiotensin-converting enzyme inhibitor, AISI = aggregate index of systemic inflammation, ARB = angiotensin receptor blocker, BMI = body mass index, CCB = calcium-channel blocker, CI = confidence interval, CRP = C-reactive protein, DBP = diastolic blood pressure, eGFR = estimated glomerular filtration rate, FBG = fasting blood glucose, HbA1c = hemoglobin A1c, HDL = high-density lipoprotein, LDL = low-density lipoprotein; LVEF = left ventricular ejection fraction, n = number of participants, NOAF = new-onset atrial fibrillation, NT-proBNP = N-terminal pro–B-type natriuretic peptide, OR = odds ratio, SBP = systolic blood pressure, TC = total cholesterol, TG = triglycerides, WBC = white blood cell.

**Table 3:** Multivariable regression analysis.

Variables	OR	95% CI	*P* value
AISI	2.335	1.532–3.560	< .001
CRP, mg/L	1.009	1.002–1.017	.015

Adjusting for AISI, WBC, CRP, eGFR, FBG, age, sex, K+, and LVEF.

AISI = aggregate index of systemic inflammation, CI = confidence interval, CRP = C-reactive protein, eGFR = estimated glomerular filtration rate, FBG = fasting blood glucose, LVEF = left ventricular ejection fraction, OR = odds ratio, WBC = white blood cell.

### 3.3. ROC analysis of NOAF

**Figure 3.**
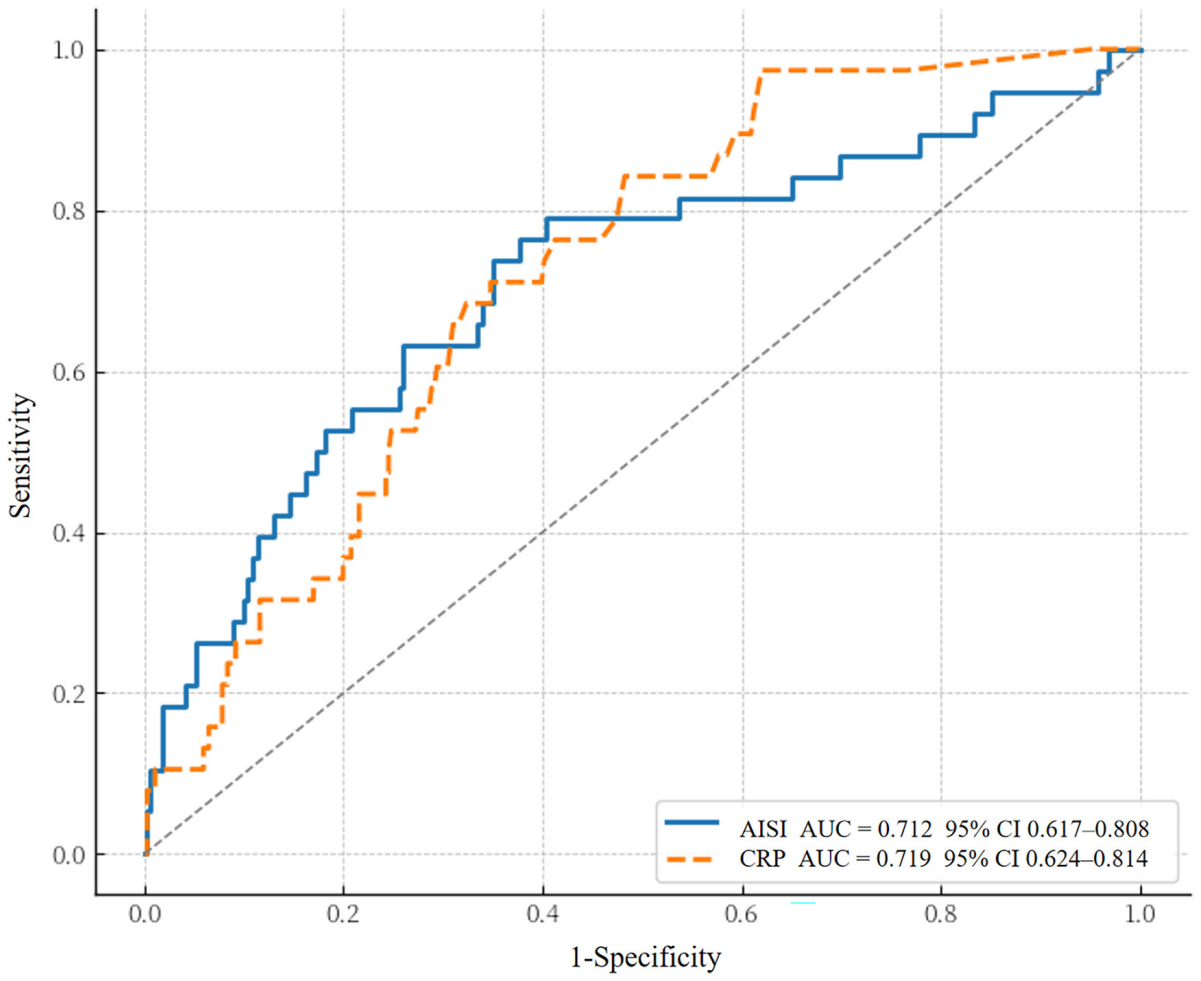
ROC analysis of inflammatory biomarkers for NOAF. AISI = aggregate index of systemic inflammation, AUC = area under the curve, CI = confidence interval, CRP = C-reactive protein, NOAF = new-onset atrial fibrillation, ROC = receiver operating characteristic.

In ROC analyses for NOAF, AISI yielded an area under the curve of 0.712 (95% CI 0.617–0.808; *P* < .001), with an optimal cutoff of 750, corresponding to a sensitivity of 0.737 (95% CI 0.569–0.866) and a specificity of 0.650 (95% CI 0.599–0.698). CRP demonstrated an area under the curve of 0.719 (95% CI 0.624–0.814; *P* < .001), with an optimal cutoff of 5.40 mg/L, yielding a sensitivity of 0.711 (95% CI 0.541–0.846) and a specificity of 0.652 (95% CI 0.601–0.701) (Fig. 3, Table 4).

**Table 4:** Receiver operating characteristic analysis for NOAF.

Variables	AUC	95% CI	*P* value	Cutoff	Sensitivity	Specificity
AISI	0.712	0.617–0.808	< .001	750	0.737	0.650
CRP, mg/L	0.719	0.624–0.814	< .001	5.40	0.711	0.652

AISI = aggregate index of systemic inflammation, AUC = area under the curve, CI = confidence interval, CRP = C-reactive protein, NOAF = new-onset atrial fibrillation.

## 4. Discussion

To our knowledge, this is the first study to investigate the relationship between AISI and NOAF in patients with MINOCA. The principal findings are as follows. First, the incidence of NOAF among patients with MINOCA was 9.3%. Second, AISI was independently associated with NOAF. Third, the unadjusted RCS analysis suggested a nonlinear dose-response relationship between AISI and NOAF, whereas this relationship was attenuated and became statistically nonsignificant after adjustment for relevant clinical covariates.

To our knowledge, reports specifically quantifying the incidence of NOAF among patients with MINOCA remain scarce. In our cohort, the incidence of NOAF was comparable to that reported in some prior studies of obstructive myocardial infarction.^[21,22]^ Although patients with MINOCA lack significant stenosis in the major epicardial coronary arteries, this does not imply a benign condition.^[2]^ Prior studies have shown that CMD plays a pivotal role.^[1,23]^ As is well recognized, effective myocardial perfusion depends more directly on the microcirculation, which may account for the findings of the present study. These observations underscore the importance of context-specific risk stratification for NOAF in MINOCA. The exploration and development of practical risk stratification tools may be of considerable value.

In recent years, AISI has garnered increasing attention as a useful inflammatory biomarker. In the MI and AF, AISI has been demonstrated to be a useful risk marker.^[13,14]^ Consistent with prior studies, our work identifies AISI as an independent risk marker for NOAF in patients with MINOCA. Although the unadjusted RCS analysis suggested a nonlinear dose-response pattern, this relationship was attenuated after adjustment for relevant clinical covariates, indicating that the nonlinear association should be interpreted cautiously. Covariates in the multivariable regression analysis included inflammatory markers such as CRP and WBC, which suggests that AISI provides additional information independent of the traditional inflammatory marker in relation to NOAF. By integrating signals from neutrophils, monocytes, platelets, and lymphocytes, AISI more comprehensively reflects activation of the inflammation-thrombosis network and, in theory, offers broader coverage than single- or dual-parameter indices.^[10–12]^ During the acute phase of MI, several pathophysiological processes are closely linked to the development of NOAF. The components of AISI play important roles throughout this cascade. In acute myocardial ischemia, neutrophils are rapidly mobilized and adhere to or infiltrate the infarcted myocardium, releasing reactive oxygen species and proteases, thereby exacerbating microvascular obstruction and reperfusion injury, and amplifying local and systemic inflammation.^[24,25]^ Platelets release thromboxane, serotonin, P-selectin, and chemotactic mediators that promote leukocyte adhesion and inflammation-coagulation coupling; platelet-leukocyte aggregates further enhance microvascular plugging.^[26,27]^ This proinflammatory-prothrombotic milieu promotes atrial injury and ischemia, thereby increasing the likelihood of atrial electrical and structural remodeling.^[28,29]^ Following AMI, circulating monocytes are mobilized for debris clearance and inflammatory amplification. This systemic monocyte activation may facilitate atrial inflammatory infiltration.^[29,30]^ Besides, acute stress commonly leads to peripheral lymphopenia, reflecting stress burden and immune disequilibrium, often accompanied by sympathetic activation and hemodynamic instability, which also can impair atrial electrical and structural remodeling.^[31]^ By multiplicatively aggregating elevated neutrophils, platelets, and monocytes, and weighting by low lymphocyte counts as an “amplifier,” AISI provides a holistic index of immune-inflammatoryactivation. Accordingly, its association with NOAF in the acute MINOCA setting appears biologically plausible. Beyond inflammation and thrombosis, oxidative stress may provide an additional mechanistic link between systemic inflammation and NOAF in MINOCA. Inflammatory-cell and platelet activation can promote reactive oxygen species generation, mitochondrial dysfunction, endothelial injury, impaired calcium handling, and altered atrial ion-channel function, thereby facilitating atrial electrical remodeling. Emerging bioelectrochemical approaches for detecting oxidative stress-related biomarkers may further complement AISI-based risk stratification in future studies.^[32,33]^ It is worth noting that although inflammatory infiltration plays an important role in atrial remodeling,^[34]^ an increased left atrial diameter did not show a statistically significant difference between the NOAF group and controls in our study. This finding may be partly attributed to the limited sample size and the single-center nature of our cohort. More importantly, structural atrial remodeling is typically a chronic process, whereas AMI is accompanied by intense inflammatory responses that may transiently alter atrial electrophysiological properties and thus increase the risk of NOAF. In summary, stronger evidence from specifically designed studies is needed to further elucidate these mechanisms. In our cohort, AISI also showed moderate discrimination for NOAF, with a cutoff of 750 balancing sensitivity and specificity. Although the level of evidence is preliminary, these results suggest a promising direction for future research. Future work should focus on prospective, multicenter validation studies incorporating standardized monitoring protocols and time-to-event analyses.

AISI offers a simple and biologically rational dimension derived from routine complete blood counts. In clinical practice, such a composite inflammatory index may be incorporated into periprocedural risk assessment and management pathways for patients with MINOCA to facilitate early identification of high-risk individuals and optimization of nephroprotective strategies. However, considering the moderate discrimination for NOAF, AISI should be positioned as an adjunct, and firm cutoff claims should be avoided.

This study also has certain limitations. First, the single-center, retrospective design limits causal inference and external generalizability, and residual confounding cannot be fully excluded. The fewer NOAF events may lead to a risk of overfitting. Second, in our study, continuous electrocardiographic monitoring was not maintained throughout the entire hospitalization, which may have led to misclassification of some AF events, particularly paroxysmal and subclinical episodes. Third, AISI was measured at a single time point and may be influenced by acute-phase inflammatory fluctuations and volume status, potentially leading to exposure misclassification; future work should incorporate time-series data to delineate longitudinal trajectories. Fourth, cardiac magnetic resonance, intravascular ultrasound, and optical coherence tomography provide valuable information for identifying the underlying causes of MINOCA. Unfortunately, as this was a retrospective study, these imaging modalities were not routinely performed for all MINOCA patients in our center, resulting in the absence of corresponding imaging evidence. Fifth, MINOCA represents a distinctive MI phenotype with complex etiologies; therefore, some of our findings may require replication in populations with obstructive MI.

## 5. Conclusions

Higher AISI is independently associated with an increased risk of NOAF in patients with MINOCA. Although the unadjusted RCS analysis suggested a nonlinear dose-response relationship, this association was attenuated and became statistically nonsignificant after adjustment for relevant clinical covariates. Given its moderate discriminative performance, AISI may serve as an adjunctive rather than a standalone risk marker, and these findings require prospective, multicenter validation.

## Author contributions

**Conceptualization:** Qian Shen, Yu Qian, Kaili Yang.

**Data curation:** Yifei Tao, Jiayu Yin, Zhixian Chen.

**Formal analysis:** Qian Shen, Yifei Tao, Jiayu Yin, Zhixian Chen.

**Investigation:** Qian Shen, Yifei Tao, Jiayu Yin, Zhixian Chen.

**Methodology:** Qian Shen, Yifei Tao, Jiayu Yin, Zhixian Chen.

**Project administration:** Yu Qian, Kaili Yang.

**Resources:** Yu Qian, Kaili Yang.

**Software:** Yu Qian, Kaili Yang.

**Supervision:** Yu Qian, Kaili Yang.

**Validation:** Qian Shen, Yifei Tao, Yu Qian.

**Visualization:** Qian Shen, Yifei Tao, Yu Qian.

**Writing** – **original draft:** Qian Shen, Yifei Tao, Jiayu Yin, Zhixian Chen, Yu Qian, Kaili Yang.

**Writing** – **review & editing:** Qian Shen, Yu Qian, Kaili Yang.
